# A risk prediction nomogram of endometrial carcinoma and precancerous lesions in postmenopausal women: A retrospective study

**DOI:** 10.1097/MD.0000000000033087

**Published:** 2023-02-22

**Authors:** Jinhua Wang, Songkun Gao, Jiandong Wang, Tong Wang

**Affiliations:** a Beijing Obstetrics and Gynecology Hospital, Capital Medical University, Beijing, China; b Beijing Maternal and Child Health Care Hospital, Beijing, China; c Beijing Youan Hospital, Capital Medical University, Beijing, China.

**Keywords:** endometrial cancer, high risk factors, nomogram, postmenopausal women, predictors

## Abstract

This study aimed to develop a risk prediction nomogram for endometrial carcinoma and precancerous lesions in postmenopausal women to provide postmenopausal patients with more information on disease probability, work out personalized medical plans, and reduce unnecessary invasive clinical examinations. We enrolled 340 patients who underwent hysteroscopy at Beijing Maternity Hospital between March 2016 and July 2018. The patients were divided into the low-risk (275 patients) and high-risk (65 patients) groups, according to the results of the pathological examinations. Binary logistic analysis was performed to evaluate the 20 potential risk factors for endometrial cancer and precancerous lesions in postmenopausal women and to screen for certain risk factors using the Statistical Package for the Social Sciences version 26.0. Using R 4.0.3, we built a prediction nomogram that incorporated the selected factors. The discrimination, calibration, and clinical usefulness of the prediction model were assessed using the concordance (C)-index, calibration plot, and decision curve analysis. Internal validation was assessed using bootstrapping validation. Predictors included in the prediction nomogram included obesity, vaginal bleeding, family history of gynecological malignancies, endometrial thickness ≥ 1.15 cm, and color Doppler flow imaging blood flow. The model displayed good discrimination, with a C-index of 0.853, and good calibration. Decision curve analysis showed that the model was clinically useful, with a benefit range of 2% to 93%. A high C-index value of 0.844 could still be reached in the interval validation. Obesity, vaginal bleeding, family history of gynecological malignancies, endometrial thickness ≥ 1.15 cm, and color Doppler flow imaging blood flow were independent risk factors for endometrial cancer and precancerous lesions. Thus, the prediction nomogram can be conveniently used to facilitate individual risk prediction in patients with endometrial cancer and precancerous lesions.

## 1. Introduction

Endometrial carcinoma (EC) is a group of epithelial malignancies usually found in perimenopausal and postmenopausal women. The incidence of EC is 10.28/100,000 according to the latest statistics of National Cancer Center in 2019.^[1]^ Thus, EC is the second most common malignant tumor of the female reproductive system after cervical cancer in China.

Screening for EC in postmenopausal women is mainly based on irregular vaginal bleeding and endometrial thickness under B-ultrasound. To prevent screening omissions, the American College of Obstetricians and Gynecologists recommends endometrial thickness ≥ 4 mm. Its negative predictive value of 99% is a reasonable substitute for sampling and is sufficient for the initial assessment of EC.^[2,3]^ However, approximately 10% to 15% of symptomatic postmenopausal women reveal an intrauterine malignancy,^[4,5]^ whereas the figure decreases to approximately 5% in asymptomatic postmenopausal women.^[6]^ This means that endometrial pathological biopsies based on postmenopausal bleeding as a single symptom and B-ultrasound results are mostly benign and are insufficient to truly predict the probability of endometrial cancer. Furthermore, it is infeasible to improve the threshold of endometrial biopsy alone, causing a reduction in sensitivity,^[7]^ but an increase in misdiagnoses. A more systematic evaluation method for postmenopausal patients from multiple perspectives should be used to further classify patients, and individualized treatment for different groups of patients should be provided, reducing the risk of invasive examination and expensive medical expenses.

A nomogram is generally used to quantitatively express the functional relationship between multiple variables using several horizontal lines. Nomograms simplify the statistical forecast model to a numerical estimate of the probability of a single even^[8]^ with its user-friendly digital interfaces, increased accuracy, and more easily understood probability of disease or prognosis,^[9]^ aiding better clinical decision-making. Therefore, nomograms are widely applied in obstetrics and gynecology related diseases for risk and prognostic assessments. ^[10–12]^

In this study, we enrolled 340 patients who underwent hysteroscopy at the Beijing Maternity Hospital between March 2016 and July 2018. As the B ultrasound truncation value in the diagnostic criteria seeks to minimize the missed diagnosis rate of patients rather than accurately indicate the disease probability of patients, we recalculated the B ultrasound truncation value, combined with other medical records, and built a risk prediction model of nomogram based on the 5 independent risk factors screened out, providing a reference for prediction and diagnosis of endometrial cancer and precancerous lesions.

## 2. Patients and methods

### 2.1. Patients

In this retrospective study, we included 340 postmenopausal women who underwent hysteroscopy at the Beijing Obstetrics and Gynecology Hospital from September 2013 to July 2018. This study was approved by the Research Ethics Committee of the Beijing Obstetrics and Gynecology Hospital (no. 2022-KY-051-01). We collected the patients’ medical history, B ultrasound findings, and endometrial biopsy results. We considered body mass index ≥ 28.0 kg/m^2^ as obesity, and we adopted the following rules as diagnostic criteria for hyperlipidemia according to the guidelines of the prevention and treatment of dyslipidemia for adults in China 2016: total cholesterol ≥ 6.2 mmol/L, triglyceride ≥ 2.3 mmol/L, low-density lipoprotein cholesterol ≥ 4.9 mmol/L, or high-density lipoprotein cholesterol ≤ 1.0 mmol/L.^[13]^ Other histories of malignancies included but were not limited to stomach and bowel cancers. Family history of diseases was collected from the patients’ immediate family members. B ultrasound examination was performed by the attending or above physician. All pathological specimens were diagnosed by pathologists who had long been involved in the diagnosis of gynecological medical records. The entire specimen was embedded in paraffin with formalin and histologically evaluated using light microscopy on hematoxylin and eosin stained slides. According to the World Health Organization classification of female reproductive organ tumors in 2014,^[14]^ precancerous lesions consist of atypical and nonatypical hyperplasia.

The inclusion criteria were as follows: Postmenopausal women, natural menopause or menopausal time ≥ 1 year; Clinical symptoms, such as vaginal bleeding and bloody secretions, or abnormal imaging examination results (the endometrial thickness measured on the largest anteroposterior diameter of the longitudinal section of the uterus was ≥ 5 mm in the ultrasound image displayed by the transvaginal ultrasound in our hospital or in an outside hospital but reexamined in our hospital); and; Hysteroscopy and endometrial biopsy performed in our hospital, with endometrial histological results obtained. The exclusion criteria were as follows: Uterine cavity space occupation revealed through imaging examination; Receiving anticoagulant or antiplatelet drug treatment; Severe cardiopulmonary insufficiency, and; Presence of acute and subacute genital tract inflammation.

### 2.2. Statistical analyses

(1) Redetermining the critical value of endometrial thickening: “Endometrial thickness” was considered the test variable, and “high risk” was considered the dependent variable. The critical value of endometrial thickness was redetermined through the receiver operating characteristic (ROC) curve using the Statistical Package for the Social Sciences (SPSS) version 26.0 (SPSS Inc., Chicago, IL).
(2) Analyzing data and evaluation: The risk prediction model of the nomogram was built based on independent risk factors screened out by binary logistic analysis (*P* < .05) using SPSS version 26.0. Calibration curves were plotted to assess the calibration of the nomograms. Harrell concordance (C)-index was used to quantify the discrimination performance of the nomogram. Decision curve analysis (DCA) was performed to determine the clinical usefulness of the nomogram by quantifying net benefits at different threshold probabilities in the cohort. The net benefit was calculated by subtracting the proportion of all patients who were false positives from the proportion of the patients who were true positives and by weighing the relative harm of forgoing interventions compared with the negative consequences of an unnecessary intervention. The prediction nomogram was subjected to bootstrapping validation (1000 bootstrap resamples) to calculate a relatively corrected C-index. All tests were conducted on both sides, and the test level was set at α = 0.05.

## 3. Results

### 3.1. Patients’ characteristics

**Figure 1.**
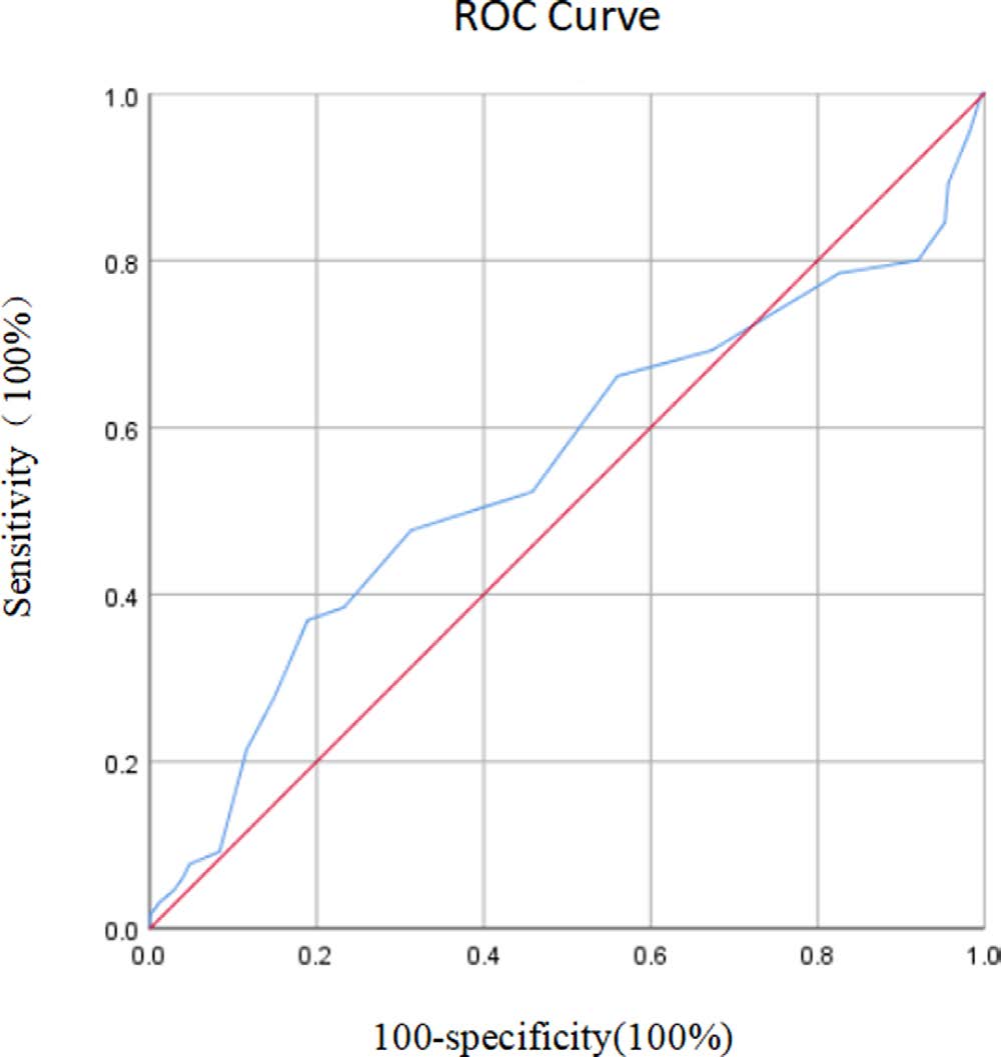
Receiver operating characteristic curve of risk for postmenopausal women with endometrial thickening. The area under the curve was 0.551 (95% confidence interval, 0.464–0.638).

This retrospective analysis included 340 patients. Based on the endometrial biopsy, we classified “endometrial cancer” and “precancerous lesions” as a “high-risk group” (n = 65) and others as a “low risk group” (n = 275). An ROC curve was drawn to identify the endometrial thickness as indicated by B-ultrasound and to identify the cutoff value of endometrial biopsy results. The cutoff value for endometrial thickness was 1.15 cm when the Youden index was maximum (Fig. 1). The characteristics of the “high risk” and “low risk” groups, including previous and present histories in the 2 groups, are presented in Table 1.

**Table 1:** Clinical data comparison between the “high risk” (n = 65) and “low risk” (n = 275) groups.

	High-risk group (65)	Low-risk group (275)	Total (340)
Characteristics	x±s		
Age (yr)	60.77 ± 6.31	59.72 ± 6.96	59.92 ± 6.85
Menopause age (yr)	50.92 ± 3.76	50.70 ± 3.63	50.74 ± 3.65
Menopausal duration (yr)	9.60 ± 7.07	8.94 ± 7.03	9.07 ± 7.04
Menarche age (yr)	14.03 ± 1.53	14.39 ± 1.91	14.32 ± 1.85
Gravidity and parity (times)	3.68 ± 2.45	4.15 ± 2.16	4.06 ± 2.23
Categorical data	n(%)		
Obesity			
Yes	18 (27.7%)	50 (18.2%)	68 (20%)
No	47 (72.3%)	225 (81.8%)	272 (80%)
Diabetes			
Yes	15 (23.1%)	51 (18.5%)	66 (19.4%)
No	50 (76.9%)	224 (81.5%)	274 (80.6%)
Hypertension			
Yes	38 (58.5%)	116 (42.2%)	154 (45.3)
No	27 (41.5%)	159 (57.8%)	186 (54.7%)
Other malignancies			
Yes	3 (4.6%)	15 (5.5%)	18 (5.3%)
No	62 (95.4%)	260 (94.5%)	322 (94.7%)
Blooding			
Yes	53 (81.5%)	79 (28.7%)	132 (38.8%)
No	12 (18.5%)	196 (71.3%)	208 (61.2%)
Family history of gynecological malignancy			
Yes	10 (15.4%)	11 (4%)	21 (6.2%)
No	55 (84.6%)	264 (96%)	319 (93.8%)
Family history of breast cancer			
Yes	1 (1.5%)	6 (2.2%)	7 (2.1%)
No	64 (98.5%)	269 (97.8)	333 (97.9%)
Family history of other malignancies			
Yes	8 (12.3%)	41 (14.9%)	49 (14.4%)
No	57 (87.7%)	234 (85.1%)	291 (85.6%)
Hormone replacement			
Yes	0 (0%)	1 (0.4%)	1 (0.3%)
No	65 (100%)	274 (99.6%)	339 (99.7%)
Tamoxifen			
Yes	0 (0%)	7 (2.5%)	7 (2.1%)
No	65 (100%)	268 (97.5%)	333 (97.9%)
Endometrium thickness ≥ 1.15cm			
Yes	32 (49.2%)	52 (18.9%)	84 (24.7%)
No	33 (50.8%)	223 (81.1%)	256 (75.3%)
CDFI blood flow			
Yes	27 (41.5%)	52 (18.9%)	79 (23.2%)
No	38 (58.5%)	223 (81.1%)	261 (76.8%)
Hysteromyoma			
Yes	26 (40%)	120 (43.6%)	146 (42.9%)
No	39 (60%)	155 (56.4%)	194 (57.1%)
Ovarian tumor			
Yes	9 (13.8%)	35 (12.7%)	44 (12.9%)
No	56 (86.2%)	240 (87.3%)	296 (87.1%)
Abnormal blood lipid			
Yes	29 (44.6%)	90 (32.7%)	119 (35.0%)
No	36 (55.4%)	185 (67.3)	221 (65.0%)

CDFI = color doppler flow imaging.

### 3.2. Feature selection

As shown in Table 2, 20 potential features were reduced to 5 predictors, including obesity (*P* = .015), vaginal bleeding (*P* < .001), family history of gynecological malignancies (*P* = .015), endometrial thickness ≥ 1.15 cm (*P* = .002), and color Doppler flow imaging (CDFI) blood flow (*P* = .005) based on the 340 patients in the cohort with binary logistic regression. The included variables of the model showed an overall statistical significance in the omnibus test (*P* < .05). The Hosmer–Lemeshow test was performed, demonstrating a good fit with a significance value of 0.773 (> 0.05).

**Table 2:** Prediction factors for endometrial cancer and precancerous lesions in postmenopausal women.

variables	b	SE	Waldχ2	*P* value	OR	95%CI
Obesity	0.124	0.051	5.964	.015	1.132	1.025–1.251
Blooding	2.802	0.428	42.794	.000	16.477	7.117–38.148
Family history of gynecological malignancy	1.606	0.659	5.934	.015	4.983	1.369–18.145
Endometrium thickness ≥ 1.15cm	1.147	0.374	9.418	.002	3.148	1.513–6.546
CDFI blood flow	1.186	0.42	7.99	.005	3.275	1.439–7.456

CI = confidence interval, CDFI = color doppler flow imaging, OR = odds ratio, SE = standard error.

### 3.3. Development of a risk prediction nomogram

**Figure 2.**
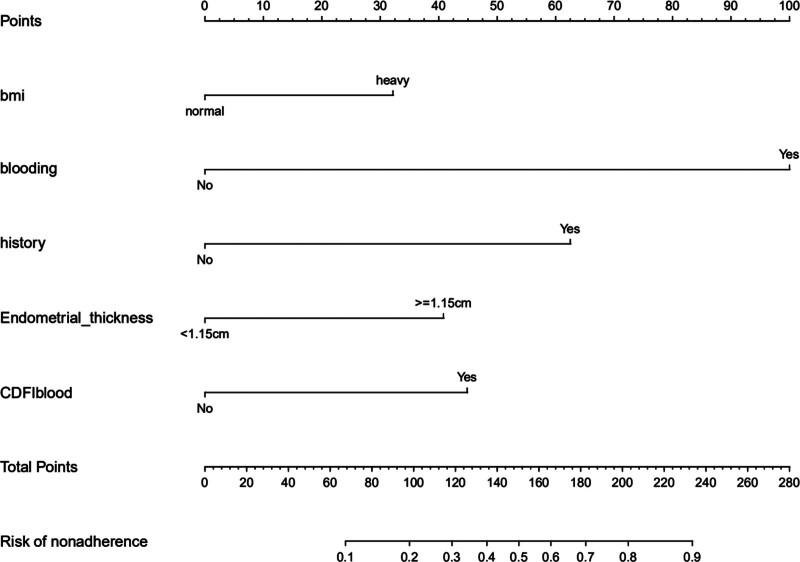
Developed risk prediction nomogram.

Based on multiple logistic regression, the model that incorporated the above independent predictors was developed and presented as a nomogram (Fig. 2), which included obesity, vaginal bleeding, family history of gynecological malignancies, endometrial thickness ≥ 1.15 cm, and CDFI blood flow. Each variable corresponded to the points on the upper scoring scale, including 32 points for obesity, 100 points for vaginal bleeding, 63 points for a family history of gynecological malignancies, 41 points for endometrial thickness ≥ 1.15 cm, and 45 points for CDFI blood flow, with a total point range of 0 to 280 points. The probability of a patient’s EC diagnosis can be obtained by projecting it onto a total score table. The higher the total score, the higher the risk of EC and precancerous lesions.

### 3.4. Evaluation of risk prediction nomogram

**Figure 3.**
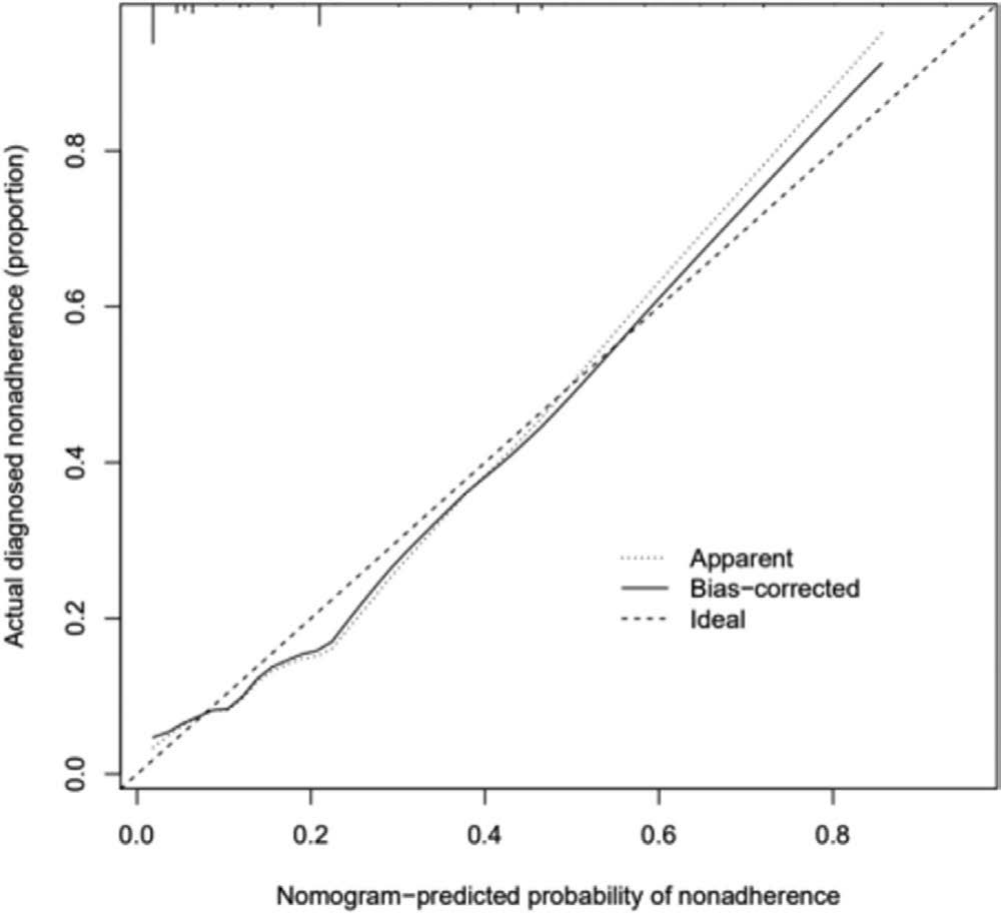
Calibration curves of the nonadherence nomogram prediction in the cohort.

**Figure 4.**
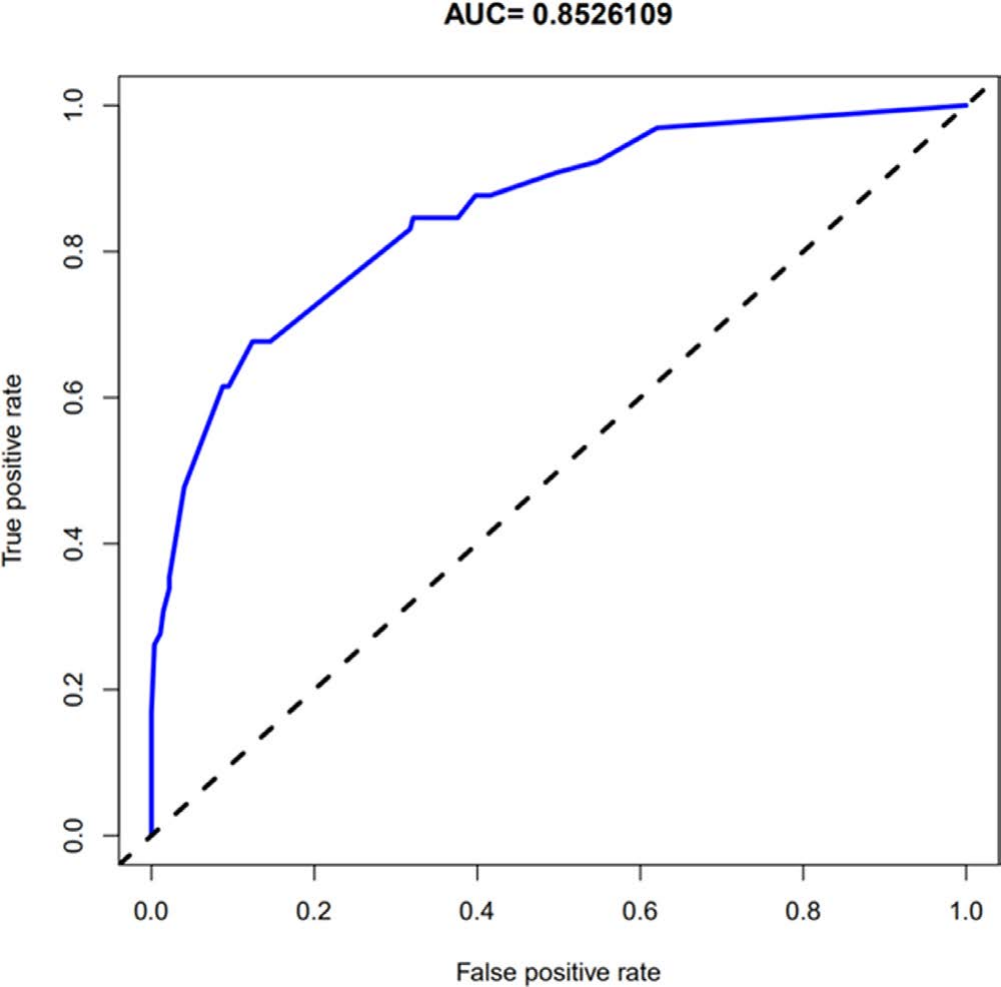
Receiver operating characteristic curve of the risk prediction nomogram.

The calibration curve of the risk prediction nomogram for the prediction of endometrial cancer and precancerous lesions indicated a good discrimination of the model. The patients in this cohort demonstrated good agreement (Fig. 3). The C-index for the prediction nomogram calculated by the ROC curve was 0.853 for the cohort, which was confirmed to be 0.844 through bootstrapping validation, suggesting good discrimination in the model (Fig. 4).

**Figure 5.**
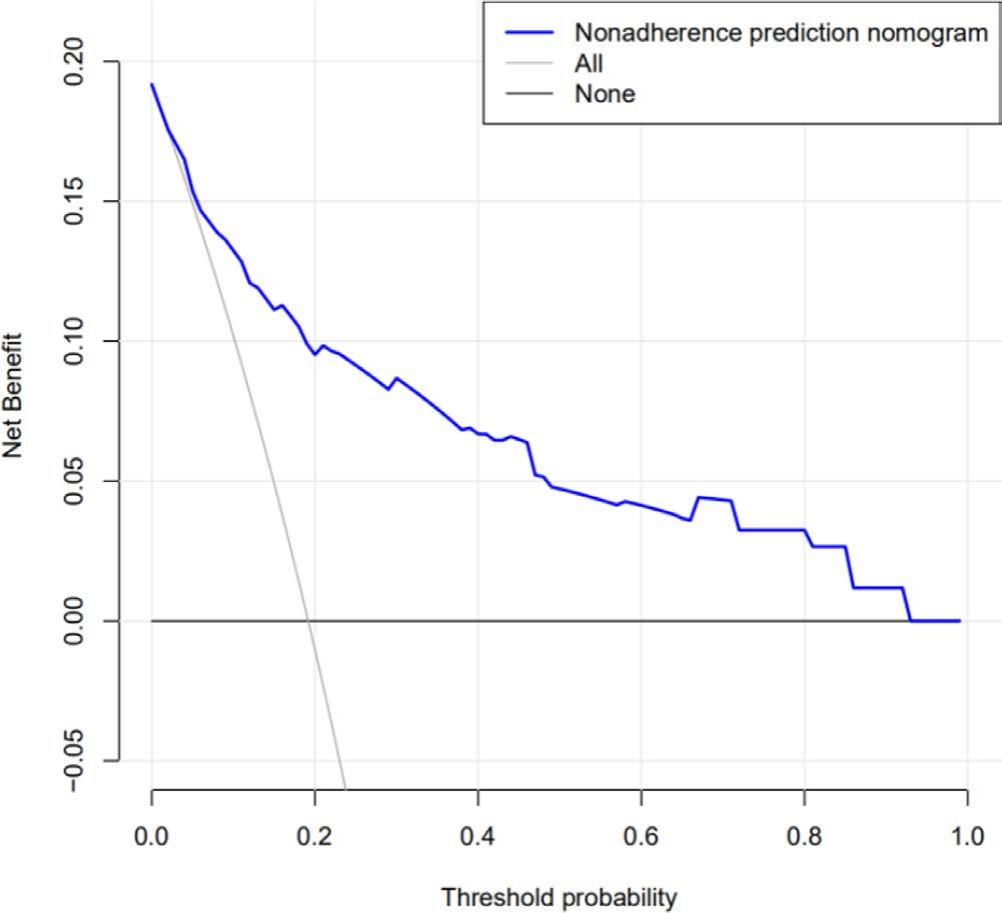
Decision curve analysis for the prediction nomogram.

The DCA of the risk prediction nomogram is shown in Figure 5. The decision curve showed that, if the threshold was between 2% and 93%, using this risk prediction nomogram to predict the risk of endometrial cancer and precancerous lesions added more benefit.

## 4. Discussion

Postmenopausal women are generally judged whether they need to undergo hysteroscopy based on their symptoms and B ultrasound results. In this study, we designed a statistical prediction model based on medical history and imaging results to quantify the probability of endometrial cancer in patients. Determining the probability of endometrial cancer in postmenopausal women can help stratify patients, allow for more accurate follow up and examination, and avoid unnecessary invasive procedures. In this study, we screened for 5 readily available predictors of endometrial cancer and precancerous lesions, including obesity, family history of gynecological malignancies, vaginal bleeding, endometrial thickness ≥ 1.15 cm, and CDFI blood flow, which have been supported in different studies. The C-index and area under the curves showed that our nomogram had good discriminative ability for the prevalence of endometrial cancer in patients.

Medical and family histories are the earliest and easiest information that physicians need to collect and cause little harm to patients. Irregular vaginal bleeding has been recognized as a hallmark symptom of endometrial cancer, Giannella et al^[15]^ study demonstrated that > 90% of patients with endometrial cancer had abnormal uterine bleeding and that < 5% of patients with endometrial cancer had no clear clinical symptoms. In addition to postmenopausal bleeding as clearly stated in the guidelines, in a prospective cohort study conducted by Kitson et al,^[16]^ family history of endometrial cancer was associated with an increased risk of endometrial cancer. If a first degree relative was diagnosed with endometrial cancer before the age of 50 years, the risk of endometrial cancer in the offspring was 6.68%. These findings were similar to those of our report on medical and family histories. We found that a history of abnormal uterine bleeding and a family history of endometrial cancer were the 2 most important factors in endometrial cancer prediction. In contrast, breast cancer history, tamoxifen use, and hormone replacement therapy were not predictors of endometrial cancer in multivariate logistic regression analysis. A history of breast cancer, tamoxifen use, and hormone replacement therapy have been considered to increase the probability of endometrial cancer in patients in several studies,^[17,18]^ but they have no effect on patients compared with other screened factors. Impressions are not sufficiently important.

Obesity is more closely associated with endometrial cancer than any other cancers. Several studies have demonstrated that obesity is an independent risk factor for endometrial cancer.^[15,19–21]^ Compared with normal weight postmenopausal women, obese women have a threefold higher risk of endometrial cancer.^[22]^ The relationship between obesity and endometrial cancer may be related to the production of aromatase in adipocytes, which promotes estrogen production, increases the levels of insulin-like growth factor 1, and induces kohalin factor secretion.^[19]^

To minimize the false negative rate of patients, guidelines suggest that hysteroscopy should be performed when endometrial thickness is > 0.5 cm, but this standard can be increased when other factors are taken into consideration. Cong et al^[23]^ found that endometrial thickness > 0.7 cm was a risk factor for atypical endometrial hyperplasia after combining other influencing factors. Through our study, we believe that the standard can be increased to > 1 cm by combining other data. However, if other data are not combined, the standard accuracy is only 81.4% (data not shown). Color Doppler ultrasound can identify new blood vessels in several tumors; because the blood supply of malignant tumors is higher than that of the general tissue, it can be used to distinguish between benign and malignant tumors. Detection of CDFI blood flow is of great significance in the diagnosis of endometrial cancer. Emoto et al^[24]^ study demonstrated that for patients with endometrial cancer, detection of intratumorally blood flow may be helpful in distinguishing low and high grade tumors and predicting myometrial invasion.

The majority of hysteroscopic biopsy results in postmenopausal women with endometrial thickening were normal tissues or benign lesions. Overactive medical interventions based only on endometrial thickening waste a large amount of healthcare resources and assume a higher risk of hysteroscopy simultaneously. Therefore, it is necessary to establish an evaluation system that can classify patients more accurately based on clinical information and reduce the frequency of invasive tests while ensuring patient safety. This study established a prediction nomogram based on these 5 independent risk factors for endometrial cancer and precancerous lesions. On the 1 hand, the prediction nomogram model had good consistency and accuracy through the ROC curve, calibration curve, DCA, and bootstrap. On the other hand, most predictors selected by the model, such as obesity, vaginal bleeding, and a family history of gynecologic malignancies, could be easily obtained during consultation. In addition, transvaginal color Doppler ultrasonography is more convenient than other expensive examinations, such as computed tomography and magnetic resonance imaging.

To the best of our knowledge, this is the first study to investigate the accuracy of history, test results, and B-ultrasound findings in the combined diagnosis of endometrial cancer by retrospectively analyzing the histological results of hysteroscopy. In this retrospective study, as almost every patient with suspected endometrial cancer underwent hysteroscopy, the selection offset should be significantly small. However, this is still a single-center study, and large prospective studies will better establish predictive models.

## 5. Limitations

This was a single center retrospective case control study with a small sample size and limited inclusion of the influencing factors. In addition, although the robustness of our nomogram was examined extensively with internal validation using bootstrap testing, external validation could not be conducted, and generalizability was uncertain for patients in other centers. These deficiencies have some effects on the accuracy of the results, which need to be externally evaluated in a wider population.

## 6. Conclusion

This study developed a novel nomogram with relatively good accuracy to help clinicians assess the risk of endometrial cancer and precancerous lesions based on the 5 easily adjusted predictors. With the help of this information, patients can know the probability of this disease more simply and effectively. This nomogram requires external validation and external evaluation in a wider population.

## Author contributions

**Conceptualization:** Wang Tong, Wang Jiandong.

**Formal analysis:** Gao Songkun.

**Investigation:** Wang Jinhua.

**Writing – original draft:** Wang Jinhua.

**Writing – review & editing:** Gao Songkun, Wang Tong, Wang Jiandong.
